# Effective Behaviors in Work Teams: Spanish Adaptation of the Individual Behavior Analysis Scale

**DOI:** 10.3389/fpsyg.2022.809731

**Published:** 2022-03-08

**Authors:** Tomas Bonavia, Martín Julián

**Affiliations:** ^1^Department of Social Psychology, Universitat de València, Valencia, Spain; ^2^Department of Psychology, Faculty of Health Sciences, Universidad Europea de Valencia, Valencia, Spain

**Keywords:** teamwork behaviors, teamwork skills, work team management, interprofessional teamwork, older adults

## Abstract

There are hardly any instruments to measure teamwork *behaviors* from an individual approach. This applies both in interprofessional teams or not, and in teams involved in health, social care, and other areas. The Individual Behavior Analysis (IBA) scale measures efficacious behavior in work teams. It is one of the few instruments proposed in the literature to measure personal skills necessary for teamwork. Only a previous exploratory analysis of the scale was informed in another study. This article analyzes its internal structure using different confirmatory factor analyses and its internal consistency, with a sample of 815 employees working for Spanish social organizations in the geriatric field, both private and public. The results of the definitive version adapted to Spanish, referred to as Individual Behavior Analysis −25, indicate a good fit of the model to the data and good reliability. Factor analysis confirmed the existence of two factors: Communication skills and Acceptance, with good internal consistency coefficients. This scale is a useful instrument for assessing, based on the reviewed literature, two of the most important individual skills an efficacious team should have.

## Introduction

What do we mean when we refer to a good “team player”? Currently, there is extensive literature about teamwork (Scholtes et al., 2003; Delgado Piña et al., 2008; West, 2012; Zaccaro et al., 2012; Salas et al., 2017), but the qualities a person needs to be considered a good team member are not very clear (Bonavia et al., 2015; Torrelles-Nadal et al., 2015). The observable individual behaviors (not considering, therefore, thoughts and attitudes) that favor collaboration within a team are still poorly defined (Varney, 1989; Leggat, 2007). There are broad lists of recommended behavioral skills (Cannon-Bowers et al., 1995; Taggar and Brown, 2001; Leggat, 2007; Shanahan et al., 2007), but few instruments that evaluate them in the specific field of interprofessional teamwork in organizations (Stevens and Campion, 1994; Harris and Barnes-Farrell, 1997; Luca and Heal, 2006; Leggat, 2007). Is it possible to have a reliable and valid instrument that assesses some of these behavioral skills, which are necessary for the proper functioning of interprofessional teams?

In this study, we aim to validate an instrument designed to measure some of the personal skills (not personality traits) necessary for teamwork. The questionnaire formulated by Harris (1995, 2001), referred to as the “Individual Behavior Analysis” (IBA), focuses on evaluating the necessary skills at an individual level to function correctly in a group or on a team (that is, it can be applied to either groups or teams). To do so, it is based both on the person’s own perception of his/her behavior and the perceptions of the other members of the team: boss or coordinator, collaborators, and subordinates (if there are any), coworkers, and other relevant figures. In sum, it consists of applying the evaluation method known as 360-degree appraisal or multisource feedback (Levy-Leboyer, 2000; Thistlethwaite et al., 2016).

The IBA questionnaire can be used in any type of context and with work teams functioning on any level, and it can also be employed in both the team creation and development processes. Likewise, it can be used as a self-assessment instrument (as a self-improvement tool) or to assess other people (in performance evaluation processes). Only one study (Bonavia et al., 2015) has measured the validity of this questionnaire in a first exploratory analysis. In the present study, we aim to confirm its internal structure through confirmatory factor analysis (CFA).

We chose this instrument because it is one of the very few tools that focus on personal skills and individual behaviors of team members. To our knowledge, there are only two instruments with a similar purpose (see Stevens and Campion, 1999; Baker et al., 2005). However, they both refer to knowledge about the required skills for working in a team. As the authors recognize (Stevens and Campion, 1999; Baker et al., 2005), the ability to respond to the items of their instruments does not necessarily reflect the behaviors that are actually performed in real situations, since knowing what to do in a given situation is quite different from the fact of actually doing it. Having discarded therefore both, we proceed to make some theoretical clarifications about the concepts involved in our research.

A related, but not completely equivalent, concept to the analysis of personal teamwork skills has to do with competences. “Teamwork” is sometimes considered a competence in itself (Aguado García et al., 2011; Torrelles-Nadal et al., 2015; Navarro et al., 2017), but this article is oriented toward the measurement of individual skills that allow people to work well on a team (Varney, 1989). Likewise, the topic of collective competences is left out for the same reason, because we are interested in the individual analysis of the necessary competences for being considered an efficacious member of a group. In addition, the concept of competences is broader than the concept of skills because it also includes knowledge, attitudes, abilities, or capabilities, and even personal motives and values (Leggat, 2007; Torrelles-Nadal et al., 2015). Finally, most of the measures that have been developed to evaluate competences in work teams use the critical incidents technique based on real jobs or carrying out specific tasks (Stevens and Campion, 1999; Taggar and Brown, 2001; Mathieu et al., 2008). Therefore, they can only be used with rigor to evaluate the competences that have to be performed successfully in these jobs or tasks. The spread of their use to other different domains for which they were not designed logically poses serious problems.

The topic of team roles also exceeds the aims of this study. The concept of role maintains a close link with the concept of status or position in a group (Ros, 2006). The group members can take on a certain set of roles, but it is not expected that everyone will assume them in the same way. Instead, the typical role analysis aims to characterize the most common pattern of behaviors in a person. For this reason, it is desirable to have instruments that are useful to evaluate aspects that are present in all the members of a team, regardless of the roles they assume. The measurement of interpersonal aspects (cooperation, cohesion, group climate, etc.) to evaluate work teams’ functioning will not be addressed in this article either (Ficapal-Cusí et al., 2014). As Stevens and Campion (1999) point out, group-level research on the processes inside work teams has been quite extensive, whereas the individual level has been relatively unattended. For this reason, it is important to develop instruments that allow us to make progress in understanding the individual factors that favor teamwork.

In recent years, a significant progress has been made to evaluate teamwork in many fields, especially in health and social care areas (Healey et al., 2006; Weaver et al., 2014; Valentine et al., 2015; Shoemaker et al., 2016; Thistlethwaite et al., 2016). Work teams composed of different professionals are a modern-day fact (Reeves et al., 2010). It is not surprising that there is an increasing development of instruments that aim to measure the different aspects involved in the interprofessional teamwork (Valentine et al., 2015; Shoemaker et al., 2016). Several of these instruments have been made to be used in specific contexts, for example, in operating rooms such as the Observational Teamwork Assessment in Surgery (OTAS) (Healey et al., 2006). Another example is the Assessment of Interprofessional Team Collaboration Scale (AITCS), created to evaluate collaboration within teams and including patients as part of the team practice (Orchard et al., 2012; Hellman et al., 2016).

Interprofessional education is another highly developing area, and it is also widely accepted worldwide as a key part of training for healthcare and other social service professionals (World Health Organization, 2010; Tamura et al., 2012; Williams et al., 2018). Despite the development of multiple measurement instruments, some authors have stated that this is still an area that requires further exploration (Archibald et al., 2014). Some of these instruments are the Interprofessional Collaborative Competency Attainment Survey (ICCAS) or the Readiness for Interprofessional Learning Scale (RIPLS).

The first one (ICCAS) was based on a set of interprofessional care competencies that students are required to self-assess: communication, collaboration, patient/family centered approach, roles and responsibilities, conflict resolution and management, and team functioning (Archibald et al., 2014). On the other hand, the RIPLS evaluates Student’s readiness to engage in interprofessional learning activities (Parsell and Bligh, 1999; Tamura et al., 2012). It is a self-report questionnaire to measure three interprofessional education readiness factors from an *attitudinal* approach: teamwork and collaboration, professional identity, and roles and responsibilities. However, although work team has become a nuclear area of study in interprofessional education (Archibald et al., 2014; Prentice et al., 2016; Ganotice and Chan, 2018; Haruta et al., 2018; Williams et al., 2018), there are hardly any instruments to measure teamwork *behaviors* from an individual approach.

There are excellent measurement instruments to assess interprofessional teamwork for the observation and feedback on a Student’s individual behavior during a team-based activity (iTOFT Consortium, 2015). According to their exhaustive review of team measures (iTOFT Consortium, 2015), which complements a review conducted by Valentine et al. (2015), none of the measures (except two, whose items were integrated in the subsequent development of this tool known as iTOFT) were made to observe individual behaviors within teams. Thus, the lack of measurement tools for observing Students’ individual behaviors while working in teams is clear (Thistlethwaite et al., 2016). This situation can be generalized not only to student training, but also to professional practice.

Enhancing team performance as a whole and having measurement instruments to assess effectiveness of a work team (and the underlying mechanisms) is crucial. However, developing instruments to assess individual behaviors of each team member is also important because it helps giving feedback on their behaviors and their relationship with team objectives. This feedback is not only necessary when students are learning to work collaboratively with other professionals (Tamura et al., 2012; Weaver et al., 2014; Thistlethwaite et al., 2016; Ganotice and Chan, 2018), but also when becomes an actual worker in an interprofessional team (Healey et al., 2006; Valentine et al., 2015; Prentice et al., 2016; Shoemaker et al., 2016; Haruta et al., 2018).

Elderly care is one of the areas in which different professionals interact and work together to deliver health and social care (Douglass, 2001; Johansson et al., 2010; Duner, 2013; Trivedi et al., 2013; Steffen et al., 2015). Older adults, who are often living with multiple chronic conditions, require well-trained interprofessional teams (composed by nurses, physicians, physiotherapists, occupational therapists, pharmacists, social workers, psychologists, dietitians, and speech therapists). Interprofessional team-based interventions have proven to be effective and efficient when implemented in the geriatric field (Johansson et al., 2010; Trivedi et al., 2013). There is also evidence that interprofessional teams constitute the best practice for treating older patients with chronic health conditions (Steffen et al., 2015). Our research will be developed in this area.

Finally, some authors state that “the vast majority of instruments in the interprofessional field are only available in English” (Vittadello et al., 2018, p. 267), so it can be concluded that there is a need for such instruments to be developed in other languages. The lack of Spanish-validated measurement instruments hinders the development of necessary skills to enhance teamwork among different professionals in Spanish-speaking countries. As with Japan (Haruta et al., 2018), Spain is an increasingly fast-aging society, and it requires a proper care system for elderly people with complex needs through an effective collaboration among different professionals.

Considering all these reasons, this study aims to:

• Choose an instrument to measure personal skills necessary for interprofessional teamwork (which can also be used with other types of teams).
• Based on its version translated into Spanish, analyze its internal structure and consistency implementing a Confirmatory Factor Analysis (CFA); and
• Propose a final version adapted to Spanish of this instrument to be used by Spanish-speaking people.

## Methods

### Participants

In all, 815 workers answered the questionnaire of Harris (1995), specifically a Spanish-translated version made by the “Centro de Estudios Ramón Areces” (Harris, 2001). These participants were chosen because the original study was designed to analyze interprofessional teamwork in elderly care organizations (both public and private), in a larger study on psychosocial factors and corruption. A training course on these topics given in different organizations (hospitals, nursing homes, primary care centers, home care, telecare, etc.) was used to recruit the participants (convenience sampling), which included nurses, physicians, social workers, care workers, physiotherapists, occupational therapists, psychologists, etc.

Participants were asked to participate in this study on a voluntary basis and their consent was orally obtained. Participants’ anonymity and confidentiality were prioritized during data collection, for this reason it was not possible to collect their personal information, such as age or gender, because in most of the training courses given the number of attendees was really small. Previously, approval from the Ethics Committee of the University of Valencia had been obtained.

This sample was randomly split into two groups to conduct two confirmatory factor analyses (CFA) and cross-validate the instrument. There were 405 participants in sample 1 and 410 participants in sample 2. The first CFA was carried out following the structure of a previous EFA (Bonavia et al., 2015) with 405 new participants (sample 1). After obtaining the results of this initial model, modifications were carefully made to conduct a second CFA. To cross-validate this second model, a new sample 2 (410 participants) was used.

### Instrument

The IBA (Individual Behavior Analysis) measures individual skills to perform well on a team, and it is based on three key ideas: the inclusion of a broad range of behaviors related to acceptance, authenticity, and empathy; simple wording to construct the items; and the application of strategies to reduce acquiescence (Harris, 1995, 2001). Originally, the scale consisted of 36 items. Participants rate their agreement with each statement on a five-point Likert scale: (1) Always, (2) Often, (3) Occasionally, (4) Seldom, and (5) Never. It takes less than 10–15 min to complete the scale.

In the original version, these items should be interpreted one by one because it was not possible to obtain summative scores that offered more general information than what was provided by the particular items (Harris, 1995, 2001). Then, an exploratory factor analysis (EFA) (Bonavia et al., 2015) showed a three-factor structure (3 items were eliminated in the previous analysis): communication skills (12 items), acceptance (13 items), and emotional expression (8 items).

### Data Analysis

Statistical analyses were performed with Mplus 7.1 (Muthén and Muthén, 2012) and SPSS 24. Following the criteria proposed by Hu and Bentler (1999), the fit indices used were Chi-Square (χ^2^), Comparative Fit Index (CFI), Root Mean Square Error of Approximation (RMSEA), and Weighted Root Mean Square Residual (WRMR). Weighted Least Squares Mean and Variance (WLSMV) was the estimator because items were treated as ordinal variables, given the low number of response categories (Finney and DiStefano, 2013; Brown, 2015).

Regarding cut-off values, the criteria suggested by Hu and Bentler (1999) and Kline (2011) were followed: CFI ≥ 0.95, and RMSEA ≤ 0.05 are good fit values; CFI ≥ 0.90, and WRMR < 0.08 are acceptable fit values. Given that WRMR cut-off values have not been studied yet (Muthén, 2013), this measure was considered with caution. Nonetheless, WRMR is useful because it assesses features of goodness-of-fit that other indices do not take into account (Muthén and Muthén, 2012).

Cronbach’s alpha was calculated in order to report a measure of reliability. However, given its limitations (Cho and Kim, 2015), the coefficient omega was also obtained to resolve these issues (Dunn et al., 2014).

## Results

Measures of sampling adequacy showed excellent values according to Bartlett’s sphericity test (χ^2^ = 8,656, df = 528, *p* < 0.001) and Kaiser-Meyer-Olkin (0.93).

As Table 1 shows, fit indices for the first confirmatory analysis (three factors) were not acceptable. After performing the second confirmatory analysis (two factors), the CFI value was good, and the RMSEA value was acceptable. Furthermore, WRMR was improved for the second model, which was consistent with the other results. Kline (2011) proposes that observed values of χ^2^ increase with the sample size. A large sample size (as in the present case) can produce a failure of the chi-square test, even when differences between observed and predicted covariances are minimal. In other words, a significant χ^2^ value is usually found when the sample size is large (Anderson and Gerbing, 1988).

**TABLE 1:** Fit indices for confirmatory analyses of the three models.

Model	χ^2^	*df*	CFI	RMSEA	WRMR
Three-factor	1735.21***	492	0.86	0.08	1.72
Two-factor	677.30***	274	0.96	0.06	1.23
One-factor	1402.60***	275	0.88	0.10	1.88

χ^2^, Chi-Square; df, degrees of freedom; CFI, Comparative Fit Index; RMSEA, Root Mean Square Error of Approximation; WRMR, Weighted Root Mean Square Residual. ***p < 0.001.

To achieve better fit indices in the second model, a factor was dropped (emotional expression) because of its low item loadings: item 6 (0.229), item 10 (−0.221), item 12 (0.328), item 22 (−0.026), item 23 (−0.173), item 28 (−0.058), item 34 (0.024), item 36 (0.297). Taking into account that the correlation between the Communication skills and Acceptance factors was 0.687, a one-factor model was carried out to ensure that the two-factor model was the most adequate representation of the data and rule out the possibility of a unidimensional model. Table 1 shows that the one-factor model’s indices were not acceptable.

Table 2 shows that item loadings were all large (range = 0.51–0.84, *p* < 0.001) and positive. Regarding the internal consistency of the scores, Cronbach’s alpha was excellent for the total scale and its subscales. The coefficient omega was also excellent for the total scale and its subscales (see Table 3).

**TABLE 2:** Means, standard deviations, and factor loadings of 25 definitive items.

Factor	Item (Original scale)	Mean	SD	Factor loadings	Recoding (see Appendix)
	3. Shows intelligence.	1.70	0.70	0.70***	3
	5. Expresses ideas clearly and concisely.	1.86	0.79	0.66***	5
	9. Thinks quickly.	1.87	0.74	0.71***	8
	11. Is persuasive, a “seller of ideas.”	2.32	0.92	0.61***	9
	15. Demonstrates high technical or professional competence.	1.73	0.74	0.76***	12
CS	17. Is able to get the attention of others.	2.05	0.85	0.66***	14
	19. Is quick to adopt new ideas.	1.95	0.75	0.78***	15
	21. Comes up with good ideas.	1.89	0.71	0.83***	17
	29. Presents ideas convincingly.	1.91	0.76	0.75***	20
	30. Responds frankly and openly.	1.55	0.64	0.64***	21
	33. Offers effective solutions to problems.	1.97	0.68	0.84***	24
	35. Talks in a way that others listen.	1.76	0.73	0.75***	25
	1. Helps others express their ideas.	1.91	0.76	0.63***	1
	2. Tries to understand the feelings (anger, impatience, rejection) that others in the group express.	1.67	0.68	0.67***	2
	4. Sympathizes with others when they have difficulties.	1.67	0.66	0.71***	4
	7. Is open to the ideas of others; looks for new ways to solve problems.	1.74	0.68	0.71***	6
	8. Is tolerant and accepting of other people’s feelings.	1.58	0.65	0.70***	7
	13. Listens and tries to use the ideas raised by others in the group.	1.81	0.76	0.57***	10
AC	14. Helps others in the group express their feelings (For example, when they are irritated or upset).	2.07	0.80	0.68***	11
	16. Is warm and friendly with those with whom he or she works.	1.42	0.62	0.69***	13
	20. Encourages others to talk about whatever is bothering them.	2.23	0.99	0.54***	16
	25. Encourages others to express their ideas before he or she acts.	2.03	0.77	0.65***	18
	26. Tries to help when others become angry or upset.	1.71	0.74	0.74***	19
	31. Is willing to compromise or change.	1.87	0.77	0.51***	22
	32. If others in the group become angry or upset, listens with understanding.	1.68	0.69	0.64***	23

CS, Communication skills; AC, Acceptance. ***p < 0.001.

**TABLE 3:** Means, standard deviations (SD), and reliability coefficients of IBA-25 (Spanish version).

Factor	Mean	SD	Cronbach’s α	Ω
CS	1.88	0.75	0.90	0.94
AC	1.80	0.74	0.86	0.90
Total	1.84	0.74	0.92	0.92

*CS, Communication skills; AC, Acceptance.*

## Discussion

In this study, we aimed to validate an instrument designed to measure some of the personal skills necessary for teamwork: the Individual Behavior Analysis (IBA). Our results supported the idea that a validated version of this instrument is reliable and useful to be implemented in a Spanish sample. Based on the results of the CFA, the first factor exactly reproduces the “Communication skills” (12 items) factor found in the EFA (Bonavia et al., 2015), and the second factor confirmed by the factorial analysis corresponds to the “Acceptance” (13 items) factor found in the EFA (Bonavia et al., 2015). The Appendix shows our proposal for a definitive IBA-25 scale in its Spanish version. Low scores on both factors (no inverted items) indicate a high mark on both Communication Skills and Acceptance.

Effective communication within teams is known to be a key factor that influences their results (Stevens and Campion, 1994; Cannon-Bowers et al., 1995; Harris and Barnes-Farrell, 1997; Baker et al., 2005; Shanahan et al., 2007; Archibald et al., 2014). Efficacious members on a team (Leggat, 2007) develop an open communication style, focusing on ideas and the decisions to make, and accepting responsibility for what they say. Expressing ideas in a clear and concise way, providing good suggestions and presenting them convincingly, and answering frankly and naturally are all elements that have been pointed out and confirmed in research on communication in teams (Torrelles-Nadal et al., 2015). All these aspects are included as items in this first factor, for which we propose the following definition:

### Communicative Skills

The set of necessary skills to communicate effectively and efficiently. Through this method, people interact with each other and the environment in order to obtain results and achieve goals. People who score low on this factor combine the indispensable skills to communicate their ideas adequately.

Regarding the second factor confirmed by the CFA, Acceptance, team members are expected to be open and receptive to information, ideas and feelings of others, and willing to consider problems from different points of view, in order to be effective (Varney, 1989). When facing complex problems, it is necessary to promote the team members’ participation in such a way that they can generate alternative solutions, making sure that all the perspectives are considered (Stevens and Campion, 1994). These ideas have been collected by different authors and given different names, but they do not always share exactly the same meaning: collaborative problem-solving (Stevens and Campion, 1994), decision-making (Cannon-Bowers et al., 1995), or participation (Taggar and Brown, 2001). Based on the items grouped in this factor, we propose the following name and definition:

### Acceptance

A behavioral disposition that consists of showing respect and tolerance toward the ideas and feelings of others. People who score low on this factor show a large variety of behaviors, such as: helping others to express themselves, being willing to understand their emotions (empathy), promote their ideas, and incorporate their contributions.

The use of the questionnaire by Harris (1995, 2001) presents several advantages for practitioners. The final version takes less than 4–5 min to complete. It can be used in education and training programs about teamwork. Because it focuses on behavior, and not personality traits or aptitudes, it is assumed that these behaviors can be modified and, therefore, learned (Varney, 1989; Aguado García et al., 2011; Torrelles-Nadal et al., 2015). In the same way, from a managerial perspective, it can be used in staff selection processes when the teamwork competence is a relevant aspect (Stevens and Campion, 1999), and for performance evaluation when teamwork is considered a basic skill among the employees (Harris and Barnes-Farrell, 1997). If organizations want to encourage teamwork, they should reward (acknowledging, remunerating, and promoting) these behaviors in their members by including them in their evaluation systems. With this instrument, and using a 360° methodology, as Harris (1995, 2001) proposes, ensures a less biased, more complete, and, therefore, more objective evaluation. In conclusion, the IBA-25 can play a major role when it comes to designing and implementing human resources practices that better fit the current context, where teamwork is surpassing individual work little by little (Delgado Piña et al., 2008; Marin Garcia and Zarate Martinez, 2008).

However, it is also necessary to mention some limitations. Harris’ questionnaire (1995, 2001) does not include some of the personal skills the literature has emphasized as favoring teamwork, such as: management, conflict resolution and negotiation at the individual level, promoting feedback, decision-making, problem-solving, or goal setting (for a more exhaustive description, see: Cannon-Bowers et al., 1995; Taggar and Brown, 2001; Leggat, 2007; Shanahan et al., 2007). On the other hand, from this research, we deduce that the necessary characteristics to be considered a good member of a team, in our case geriatric interprofessional teams, cannot necessarily be extrapolated to other types of teams (Taggar and Brown, 2001; Shanahan et al., 2007). We propose, however, that the two factors that make up the IBA-25 are important for considering someone an effective member of any team, regardless of the type of team or the task to be performed, although future research will have to confirm this point. In addition, in the literature it has been proposed that the necessary qualities to work efficaciously on a team probably vary across different cultures (Baker et al., 2005), which means that the questionnaire has to be subjected to cross-cultural research in order to prove its real potential. Finally, this article did not have the objective of relating to what degree the factors found in the IBA-25 at an individual level are useful in explaining teams’ performance at a collective level. This question, which might seem to be a limitation of our work, provides a great opportunity for future research.

As several studies show (Varney, 1989; Stevens and Campion, 1994, 1999; Cannon-Bowers et al., 1995; Delgado Piña et al., 2008), teamwork skills are essential for the efficacious performance of workers in many jobs today. However, in spite the progress made, we do not know what these skills are and how to measure them. The IBA-25 offers the chance to analyze individual behaviors, grouped in two sets of teamwork skills that can now be better evaluated.

## Data Availability Statement

The raw data supporting the conclusions of this article will be made available by the authors, without undue reservation.

## Ethics Statement

The studies involving human participants were reviewed and approved by the Ethics Committee of the University of Valencia. Written informed consent for participation was not required for this study in accordance with the national legislation and the institutional requirements.

## Author Contributions

TB contributed to the conception and design of the study, organized the database, and wrote the first draft of the manuscript. MJ performed the statistical analysis. TB and MJ gave the final approval of the manuscript before the submission. Both authors reviewed it critically.

## Conflict of Interest

The authors declare that the research was conducted in the absence of any commercial or financial relationships that could be construed as a potential conflict of interest.

## Publisher’s Note

All claims expressed in this article are solely those of the authors and do not necessarily represent those of their affiliated organizations, or those of the publisher, the editors and the reviewers. Any product that may be evaluated in this article, or claim that may be made by its manufacturer, is not guaranteed or endorsed by the publisher.

## Appendix

### IBA-25 (versión adaptada al español)

#### Análisis del comportamiento individual en los equipos de trabajo

Denominación del equipo: _____________________

Nombre de la persona que está usted describiendo: ____________________________________

La persona que está describiendo es: (marque una opción)

□ yo mismo/a

□ mi jefe/a

□ mi subordinado/a

□ mi compañero/a (del mismo nivel jerárquico)

□ otro caso (por favor, especifique) _______________________

***Instrucciones:*** A continuación, ofrecemos veinte y cinco descripciones de maneras en las que las personas participan en las reuniones de grupo. Para cada ítem, marque la alternativa que mejor refleje cómo actúa en las reuniones la persona que está describiendo.

Tenga en cuenta que está describiendo el comportamiento de esta persona en las reuniones, y no cómo usted la ve actuar en otros escenarios.
